# UBE2S promotes gastric cancer progression through RACGAP1 stabilization associated with K292-dependent K63-linked ubiquitination

**DOI:** 10.1080/15384047.2026.2737476

**Published:** 2026-10-08

**Authors:** HeFei Cheng, Ziqing Zhou, Qirun Cheng, Ziqi Sui

**Affiliations:** a Department of Gastroenterology, The First People’s Hospital of Linping District, Hangzhou, Zhejiang Province, People's Republic of China; b Department of Gastroenterology, The Second Affiliated Hospital of Zhejiang Chinese Medical University, Hangzhou City , Zhejiang Province, People's Republic of China; c Department of Gastroenterology, Zhejiang Hospital, Hangzhou, People's Republic of China

**Keywords:** Gastric cancer, UBE2S, RACGAP1, K63-linked polyubiquitination, protein stability

## Abstract

**Objective:**

Gastric cancer (GC) remains highly lethal, and effective molecular targets are limited. This study investigated the expression, exploratory survival associations, and mechanistic role of UBE2S in GC.

**Methods:**

UBE2S expression and survival associations were analyzed using public datasets, whereas UBE2S protein expression was assessed in seven paired GC tissues and GC cell lines. Loss- and gain-of-function assays, xenograft models, quantitative proteomics, genetic rescue, JNK inhibition, co-immunoprecipitation, cycloheximide chase, proteasome inhibition, and linkage-specific ubiquitination assays were performed.

**Results:**

UBE2S was upregulated in GC and showed apparent within-dataset discriminatory ability at the tissue-transcript level (AUC = 0.909); higher expression showed an exploratory association with unfavorable survival in public datasets. Protein assessment in seven paired tissues was consistent with this expression pattern. UBE2S depletion suppressed GC cell viability, clonogenicity, migration, and xenograft growth. Proteomics prioritized RACGAP1 as a candidate downstream effector, and subsequent rescue experiments supported its functional relevance. UBE2S associated with RACGAP1 and increased RACGAP1 protein stability without altering its mRNA expression. UBE2S depletion decreased K63-linked and increased K48-linked ubiquitination of RACGAP1, whereas MG132 partially restored RACGAP1 abundance. K292 mutation impaired UBE2S-dependent K63-linked ubiquitination, RACGAP1 accumulation, JNK/c-Jun activation, and malignant phenotypes, although RACGAP1-K292R retained partial activity. Pharmacological JNK inhibition with SP600125 suppressed RACGAP1-mediated JNK/c-Jun reactivation and clonogenic rescue.

**Conclusion:**

Our findings support a model in which UBE2S promotes GC progression by stabilizing RACGAP1 in association with K292-dependent K63-linked ubiquitination, providing a mechanistic basis for further preclinical investigation of the UBE2S/RACGAP1/JNK/c-Jun axis.

## 1. Introduction

Gastric cancer (GC) remains a major global health challenge, ranking as the fifth most frequently diagnosed malignancy with approximately 968,350 new cases and the fifth leading cause of cancer-related mortality with 659,853 deaths estimated worldwide in 2022.^1^ Despite advancements in surgical techniques and systemic chemotherapy, the five-year survival rate for patients with advanced or metastatic GC remains low, at approximately 6%.^2^

In the era of precision medicine, targeted therapies have offered significant hope; however, their clinical efficacy is frequently hampered by primary or acquired resistance. A recent perioperative study (NEOSUMMIT-02) underscored that although the combination of trastuzumab and immunotherapy demonstrates potent anti-tumor activity in HER2-positive GC,^3^ ^,^ ^4^ the enrichment of regulatory T cells (Tregs) and the loss of HER2 expression during treatment remain predominant barriers leading to therapeutic resistance.^5^ Similarly, despite the emergence of promising novel targets such as Claudin 18.2 (CLDN18.2) and the approval of zolbetuximab,^6^ this biomarker often exhibits significant spatial and temporal heterogeneity, characterized by divergent expression profiles across different time points and biopsy sites. Coupled with the current lack of long-term, standardized diagnostic criteria, these factors pose substantial challenges for precise clinical decision-making.^7^ Consequently, there is an urgent need to identify novel molecular drivers and decipher the underlying biochemical mechanisms to develop more resilient and effective intervention strategies.

Ubiquitination, the second most prevalent post-translational modification (PTM) after phosphorylation, orchestrates nearly 80%–90% of cellular proteolysis and functions as a master regulator of various cancer hallmarks.^8^ ^,^ ^9^ This dynamic process, involving a cascade of E1-E2-E3 enzymes and reversible deubiquitinases (DUBs), dictates protein fate through distinct ubiquitin linkages. Beyond the canonical K48-linked degradation, distinct ubiquitin linkages, including K11, K63, and M1, can encode diverse cellular outcomes involving proteasomal degradation, signal transduction, DNA repair, and protein scaffolding.^10^ In tumor metabolic reprogramming, the UPS frequently modulates the “Warburg effect”; for instance, the E3 ligase Parkin and the DUB OTUB2 coordinately regulate the stability of PKM2 to drive glycolysis in colorectal cancer, while the METTL5/USP5/c-Myc axis accelerates glucose metabolism in hepatocellular carcinoma.^11-13^ Furthermore, ubiquitination is indispensable for tumor immune evasion within the tumor microenvironment (TME). Emerging evidence shows that E3 ligases such as FBXO38^14^ and *β*-TRCP^15^ promote the degradative ubiquitination of PD-1 and PD-L1, respectively, whereas DUBs like USP2^16^ and USP7^17^ stabilize these checkpoints to blunt anti-tumor immunity. Additionally, the crosstalk between ubiquitination and other PTMs—such as the AKT-mediated phosphorylation of RNF12 or the BCKDK-mediated phosphorylation of STUB1—further complicates the oncogenic signaling landscape by fine-tuning substrate stability and localization.^18^ ^,^ ^19^ Collectively, these multifaceted roles underscore the therapeutic potential of targeting the ubiquitin system to overcome treatment resistance and improve clinical outcomes in malignancies like GC.

Ubiquitin-conjugating enzymes (E2s) function as important regulators within the ubiquitin-proteasome system (UPS). By orchestrating the assembly of specific ubiquitin chain topologies—ranging from degradative K11 and K48 linkages to non-proteolytic K63 and M1 linkages—E2s precisely modulate substrate stability, subcellular localization, and interactome dynamics, thereby driving the fundamental hallmarks of cancer.^20^ Dysregulation of various E2 family members has been intimately linked to malignant transformation and progression. For instance, UBE2C is frequently overexpressed in numerous solid tumors and cooperates with the APC/C complex to accelerate mitotic progression and tumor invasion.^21^ UBE2T has been shown to facilitate the polyubiquitination and degradation of the core tumor suppressor p53, thereby impairing apoptotic signaling.^22^ Furthermore, UBE2D3 exhibits functional pleiotropy, contributing to immune evasion in pancreatic cancer by inhibiting antigen presentation pathways.^23^ Other family members, such as UBE2M, promote oncogenic cell cycle progression via neddylation,^24^ while UBE2O orchestrates metabolic reprogramming by mediating the degradation of the energy sensor AMPKα2.^25^ As a prominent Class I E2 enzyme, ubiquitin-conjugating enzyme E2 S (UBE2S) has emerged as an important factor implicated in the maintenance of chromosomal instability and the remodeling of the immunosuppressive tumor microenvironment.^26^ ^,^ ^27^ However, despite its established roles across a broad spectrum of malignancies, the precise biochemical mechanisms and the specific signaling circuits hijacked by UBE2S in the progression of GC remain largely incompletely understood and have not been comprehensively investigated.

In the present study, we investigated the expression profile, survival associations, and biological role of UBE2S in GC. Through quantitative proteomics and biochemical validation, we prioritized RACGAP1 as a downstream effector and UBE2S-associated protein. Our findings support a model in which UBE2S promotes K292-dependent K63-linked ubiquitination and stabilization of RACGAP1. Direct comparison of RACGAP1-WT and RACGAP1-K292R further demonstrated that K292 is required for the full downstream JNK/c-Jun activation and maximal increases in cell viability, clonogenic growth, and migration in response to UBE2S. Furthermore, pharmacological JNK inhibition suppressed RACGAP1-mediated JNK/c-Jun reactivation and clonogenic rescue, supporting JNK/c-Jun as a functionally relevant downstream mediator of RACGAP1.

## 2. Materials and methods

### 2.1. Bioinformatics data acquisition and pre-processing

To characterize the expression patterns of UBE2S (ENSG00000108106.14) and RACGAP1 (ENSG00000257331.1) in malignancies, high-throughput RNA-sequencing data (STAR-generated TPM format) for 33 cancer types were systematically retrieved from the Genomic Data Commons (GDC) portal (https://portal.gdc.cancer.gov). For the primary investigation into GC, the TCGA-STAD (Stomach Adenocarcinoma) cohort was prioritized. To ensure a robust normal-to-tumor baseline for pan-cancer comparisons, transcriptomic data from the Genotype-Tissue Expression (GTEx) database were integrated where necessary. For both pan-cancer cohorts and individual datasets, UBE2S and RACGAP1 expression matrices were normalized using a log_2_(TPM + 1) transformation to minimize heteroscedasticity. To maintain the stringency of downstream survival and correlation analyzes, samples lacking definitive clinical follow-up or redundant genomic entries were rigorously excluded. All computational workflows, including the extraction of UBE2S and RACGAP1 expression profiles and subsequent data visualization, were implemented within the R environment (version 4.2.1) utilizing the ggplot2 (v3.4.4), stats (v4.2.1), and car (v3.1-0) packages. The specific data processing and visualization procedures were conducted via the Xiantao Academic platform (https://xiantaozi.com/).

### 2.2. Expression profile and exploratory ROC analysis

The transcriptomic landscape of UBE2S across 33 malignancies was evaluated using the Wilcoxon rank-sum test. For the TCGA-STAD cohort, differential expression between tumor and normal tissues was assessed via the Welch t-test. Furthermore, a paired-sample t-test was employed to characterize UBE2S levels in specifically matched tumor and adjacent non-tumor tissue pairs. The ability of UBE2S expression to distinguish GC from normal tissue samples within the analyzed public datasets was explored using receiver operating characteristic (ROC) curve analysis with the pROC package (v1.18.0). The area under the curve (AUC) was reported as a measure of within-dataset discrimination; no external diagnostic validation was performed. The bioinformatic implementation and corresponding statistical analyzes were carried out through the Xiantao Academic website (https://xiantaozi.com/).

### 2.3. Evaluation of tumor immune microenvironment (TIME)

Immune infiltration levels within the GC microenvironment were quantified using the single-sample gene set enrichment analysis (ssGSEA) algorithm via the GSVA package (v1.46.0). We utilized the validated marker sets for 24 immune cell subtypes as described by Bindea et al. (2013). These cell types included activated dendritic cells (aDCs), B cells, CD8^+^ T cells, cytotoxic cells, and various T helper subsets. Samples were categorized into high- and low-UBE2S expression groups, and the variations in immune enrichment scores were compared using Student’s t-test. The immune-related bioinformatic workflows were executed on the Xiantao Academic platform (https://xiantaozi.com/).

### 2.4. Exploratory kaplan–meier survival analysis

The associations of UBE2S and RACGAP1 expression with survival outcomes in GC were evaluated using the Kaplan-Meier Plotter (https://kmplot.com). Because no clinically validated expression cutoff has been established for UBE2S or RACGAP1 in GC, the “auto-select best cutoff” function was used for the primary exploratory analysis. Survival endpoints included Overall Survival (OS), Progression-Free Interval (PFI), or Post-Progression Survival (PPS). Notably, the GSE62254 dataset was excluded from this meta-analysis due to its distinct clinical characteristics (extended survival times and shifted expression profiles) compared to other integrated cohorts, to reduce between-cohort heterogeneity.

### 2.5. Cell culture and lentiviral transduction

Human GC cell lines AGS (Cat No. SCSP-5262), HGC-27 (Cat No. TCHu 22), MKN-1 (TCHu282), MKN-74 (Cat No. TCHu284), and the immortalized gastric epithelial cell line GES-1 (Cat No. GNHu65) were obtained from *the Cell Bank of the Chinese Academy of Sciences*, Shanghai, China. NUGC-3 cells (Cat No. CTCC-007-0391) were purchased from *Meisen Cell Co., Ltd.* (Hangzhou, China). All cell lines were authenticated by short tandem repeat (STR) profiling by *Biomedical Science* (Shanghai, China) and used within 10 passages after thawing. Mycoplasma contamination was routinely tested using a luminescence-based mycoplasma detection assay (Beyotime, Cat No.C0297S), and all cell lines used in this study were confirmed to be mycoplasma-negative. Cells were maintained in RPMI 1640 or DMEM (Gibco, USA) supplemented with 10% fetal bovine serum (FBS) and 1% penicillin/streptomycin in a humidified atmosphere containing 5% CO₂ at 37 °C.

To achieve stable gene silencing or overexpression, lentiviral infection and plasmid transfection were performed. All lentiviruses and plasmids were constructed and validated by Genomeditech (Shanghai, China). For the knockdown of UBE2S, lentiviral vectors harboring specific shRNA sequences (shUBE2S-1#: ACATCATCCGCCTGGTGTA; shUBE2S-2#: CATATGCTGGAGGTCTGTT) or a negative control sequence (shCtrl: CAACAAGATGAAGAGCACCAA) were used to infect AGS or HGC-27 cells. Stable cell lines were established following selection with 2 μg/mL puromycin for one week. For RACGAP1 overexpression, the full-length RACGAP1 cDNA was cloned into expression plasmids and transfected into GC cells. To delineate the ubiquitination sites of RACGAP1, wild-type (WT) and various lysine-to-arginine (K-R) mutant plasmids (K122R, K292R, K423R, K490R, and K587R) were constructed with an *N*-terminal 3 × Flag tag. The efficiency of knockdown or overexpression was systematically verified via qRT-PCR and Western blot analysis.

### 2.6. Immunoblotting

A total of 7 paired fresh GC tissues and adjacent non-tumor tissues were obtained from patients who underwent surgical resection at the Second Affiliated Hospital of Zhejiang Chinese Medical University. These specimens were used for preliminary protein-level assessment of UBE2S expression and were not intended as an independent diagnostic or prognostic validation cohort. Tissues were snap-frozen in liquid nitrogen and stored at −80 °C. For protein extraction, tissues and PBS-washed GC cells were lysed in chilled RIPA buffer (Beyotime, Cat No. P0013B) containing protease and phosphatase inhibitors (Beyotime, Cat No. P1045). After centrifugation (12,000 g, 15 min, 4 °C), protein concentrations were determined by BCA assay (Beyotime, Cat No. P0012). Subsequently, proteins were denatured (95 °C, 5 min), resolved via SDS-PAGE, and transferred to 0.22-μm PVDF membrane. Following blocking in 5% milk, membranes were incubated overnight at 4 °C with primary antibodies (Proteintech, Wuhan, China) against UBE2S (Cat No. 14115-1-AP), RACGAP1 (Cat No. 13739-1-AP), JNK (Cat No. 51153-1-AP), *p*-JNK (Cat No. 80024-1-RR), c-Jun (Cat No. 24909-1-AP), *p*-c-Jun (Cat No. 80086-1-RR), Cyclin D1 (Cat No. 60186-1-Ig), CDK6 (Cat No. 14052-1-AP), Flag (Cat No. 66008-4-Ig), HA (Cat No. 51064-2-AP), Tubulin (Cat No. 66031-1-Ig), *β*-actin (Cat No. 66009-1-Ig), and Vinculin (Cat No. 26520-1-AP). After TBST washes and 1-h secondary antibody incubation, protein bands were visualized using ECL (Biosharp, Cat No. BL523A) and quantified using Image J.

### 2.7. Quantitative real-time PCR (qRT-PCR)

Total RNA was isolated from GC cells using the RNA-Quick Purification Kit (Yishan, Cat No. ES-RN001) according to the manufacturer’s protocol. Subsequently, complementary DNA (cDNA) was synthesized from 1 μg of total RNA utilizing the Fast All-in-One RT Kit (Yishan, Cat No. ES-RT001). Quantitative PCR was executed on an ABI 7500 Fast Real-Time PCR System with Super SYBR Green qPCR Master Mix (Yishan, Cat No. ES-QP002). Relative mRNA expression levels were normalized to GAPDH and calculated using the 2^−ΔΔCt^ method. The specific primer sequences for UBE2S and RACGAP1 are detailed in Table S1.

### 2.8. Cell viability and colony formation assays

Cell viability was evaluated using the Cell Counting Kit-8 (CCK-8; Beyotime, Cat No. C003). Briefly, AGS and HGC-27 cells (3 × 10^3^ cells/well) were seeded into 96-well plates. At the indicated time points (0, 24, and 48 h), 10 μL of CCK-8 reagent was added to each well, followed by incubation for 2 h at 37 °C. The optical density (OD) was measured at 450 nm using a microplate reader. For the colony formation assay, cells were seeded in 6-well plates at a density of 1,000 cells per well and cultured for 14 days. After visible colonies had formed, they were fixed with 4% paraformaldehyde and stained with 0.1% crystal violet. The number of colonies was manually counted and photographed.

### 2.9. Wound healing and transwell migration assays

Cell motility was assessed using wound healing and Transwell migration assays. For the wound healing assay, cells were grown to 90% confluence in 6-well plates, and a linear wound was created using a sterile 200 μL pipette tip. The cells were then washed with PBS and cultured in serum-free medium. Images of the wound area were captured at 0 and 24 h using an inverted microscope, and the migration rate was calculated as follows: (initial wound width - final wound width)/initial wound width × 100%. For the Transwell assay, 5 × 10^4^ cells in 200 μL of serum-free medium were placed into the upper chambers (8 μm pore size; Corning, Cat No. 3422), while 600 μL of medium containing 10% FBS was added to the lower chambers. After 24 h of incubation, migrated cells on the lower surface were fixed and stained with crystal violet. The number of migrated cells was then manually counted, and representative images were acquired.

### 2.10. In vivo xenograft model

Male BALB/c nude mice aged 4–6 weeks were purchased from Hangzhou Qizhen Laboratory Animal Technology Co., Ltd. and maintained under specific pathogen-free conditions at the Laboratory Animal Center of Zhejiang Chinese Medical University. Mice were housed under controlled temperature and humidity with a 12-h light/dark cycle and had free access to food and water. All mice were allowed to acclimatize to the animal facility for one week before tumor cell inoculation. The experimental unit for all animal experiments was the individual mouse. The body weight of mice at the start of the experiment was approximately 18−19 g.

Two subcutaneous xenograft experiments were performed using a total of 33 mice. For the UBE2S knockdown model, 18 mice were randomly allocated into three groups using a random number method: shCtrl, shUBE2S-1#, and shUBE2S-2# groups, with 6 mice in each group. Approximately 8 × 10^6^ HGC-27 cells stably expressing the corresponding constructs were subcutaneously injected into the right flank of each mouse. For the in vivo rescue experiment, 15 mice were randomly divided into three groups: shCtrl, shUBE2S, and shUBE2S + oeRACGAP1 groups, with 5 mice in each group. The same number of engineered HGC-27 cells was injected subcutaneously using the same procedure.

To minimize potential confounders, mice in different groups were maintained under identical housing conditions, and tumor cell injection, tumor measurement, and endpoint collection were performed according to standardized procedures. Tumor dimensions were measured every 7 days using calipers by investigators blinded to group allocation. Tumor volume was calculated as follows: volume = length × width^2^/2. The primary in vivo outcome measures were tumor growth kinetics, tumor volume, and terminal tumor weight. The sample size was determined based on preliminary experiments and commonly used sample sizes in xenograft studies; no formal a priori power calculation was performed.

Predefined humane endpoint criteria included severe body weight loss, tumor ulceration, impaired mobility, or signs of obvious distress. No animals were excluded from the final analysis unless they met the predefined humane endpoint criteria. On day 35 after tumor cell inoculation, mice were euthanized by CO₂ inhalation using a gradual-fill method at a displacement rate of approximately 30%–70% of the chamber volume per minute, followed by cervical dislocation as a secondary physical method. Death was confirmed by the absence of heartbeat and respiration. No unexpected adverse events, including spontaneous death, tumor ulceration, severe infection, or marked body weight loss, were observed during the study period. No animals met the predefined humane endpoint criteria before the scheduled endpoint.

### 2.11. 4D-DIA quantitative proteomic profiling

To systematically delineate the proteomic alterations induced by UBE2S deficiency, total protein was harvested from HGC-27 cells stably expressing shUBE2S or shCtrl. Three independent biological replicates were analyzed for each group (*n* = 3 per group), with each replicate derived from an independently cultured cell preparation. Samples were processed by Shanghai Applied Protein Technology (Shanghai, China). Proteins were extracted using SDT buffer (4% SDS, 100 mM Tris-HCl, pH 7.6) and quantified using the BCA assay. Proteins were reduced with DTT, alkylated with IAA, and digested with trypsin overnight. The resulting peptides were desalted using C18 cartridges, reconstituted in 0.1% formic acid, and supplemented with iRT reference peptides before LC-MS/MS analysis. Peptides were separated using an Evosep One system and analyzed on a Bruker timsTOF mass spectrometer using DIA-PASEF acquisition. The MS and MS/MS scan range was 100–1700 m/z, and 21 DIA acquisition windows were used. DIA data were processed using Spectronaut 19 with dynamic iRT retention-time prediction, MS2-level interference correction, and cross-run normalization enabled. Protein identification was filtered using a Q-value cutoff of 0.01, corresponding to an identification-level FDR of <1%. For the exploratory differential-abundance analysis, candidate differentially abundant proteins were defined as proteins with a fold change (FC) > 1.5 or < 0.67 together with a nominal Student’s t-test *P* < 0.05.

### 2.12. Functional annotation and pathway enrichment analysis

To translate the list of candidate differentially abundant proteins into biological insights regarding UBE2S-mediated oncogenesis, we performed Gene Ontology (GO) and Kyoto Encyclopedia of Genes and Genomes (KEGG) analyzes. This process categorized proteins according to biological processes (BP), molecular functions (MF), and cellular components (CC). Subcellular localization was predicted using CELLO. GO functional annotation was performed using Blast2GO, whereas KEGG pathway annotation was performed using KOBAS 3.0. Enrichment analysis was performed using Fisher’s exact test, and a nominal *P* < 0.05 was considered to indicate significant enrichment in the original exploratory analysis.

### 2.13. Threshold-free gene set enrichment analysis (GSEA)

Recognizing that significant biological shifts often occur through subtle, coordinated changes in entire gene sets rather than isolated high-fold-change proteins, we performed Gene Set Enrichment Analysis (GSEA). Unlike traditional enrichment which relies on a pre-defined DEP list, GSEA considers the entire proteomic spectrum. All identified proteins were ranked according to their log_2_(fold change) values between the shUBE2S and control groups. This ranked list was then interrogated against the KEGG and GO-BP libraries. A normalized enrichment score (NES) was calculated for each gene set, and statistical significance was defined by a Nominal *P* < 0.05 and a False Discovery Rate (FDR) *q* < 0.25. This approach allowed us to identify global cellular programs, such as the cell cycle and DNA replication, that are coordinately suppressed upon UBE2S silencing.

### 2.14. Correlation analysis of UBE2S and RACGAP1

The co-expression relationship between UBE2S and RACGAP1 mRNA levels was assessed using Spearman’s rank correlation coefficient (R). Utilizing the normalized TCGA-STAD expression profiles described in Section 2.1, this non-parametric analysis was executed to evaluate the monotonic association between the two transcripts. The resulting correlation was visualized as a scatter plot with a fitted linear regression line using the ggplot2 package. Statistical significance was defined by a *P*-value < 0.05.

### 2.15. Co-immunoprecipitation (Co-IP) and western blotting

Endogenous or exogenous protein associations were evaluated using the Classic Magnetic Protein A/G IP/Co-IP Kit (Yamei, Shanghai, China; Cat No. YJ201). Briefly, cells were harvested and lysed in the provided ice-cold lysis buffer supplemented with a protease inhibitor. The supernatants were collected by centrifugation at 12,000 g for 15 min at 4 °C, and then pre-cleared with Protein A/G magnetic beads for 1 h to minimize non-specific binding. Subsequently, equal amounts of protein lysates were incubated with specific primary antibodies (anti-UBE2S, anti-RACGAP1, or anti-Flag) overnight at 4 °C with gentle rotation. The resulting immunocomplexes were captured by Protein A/G beads for 4 h, followed by five washes with wash buffer. Finally, the beads were resuspended in loading buffer and boiled for 5-10 min to elute the bound proteins for downstream Western blot analysis.

### 2.16. Protein stability assay

To determine the half-life of the RACGAP1 protein, GC cells stably overexpressing UBE2S or those transfected with an empty vector were treated with 20 μg/mL cycloheximide (CHX; MedChemExpress, NJ, USA; Cat No. HY-12320) for the indicated time intervals (0, 4, and 8 h) to halt de novo protein synthesis. Total cellular proteins were extracted at each time point, and the degradation kinetics of RACGAP1 were quantitatively analyzed via Western blot.

### 2.17. Cellular ubiquitination assay

To determine the effects of UBE2S depletion on endogenous RACGAP1 ubiquitination and proteasomal turnover, AGS and HGC-27 cells stably expressing shCtrl, shUBE2S-1#, or shUBE2S-2# were treated with vehicle or 5 μM MG132 for 6 h before harvesting. Whole-cell lysates were analyzed by Western blotting for UBE2S and RACGAP1 expression. In parallel, equal amounts of total protein were subjected to immunoprecipitation using an anti-RACGAP1 antibody, followed by immunoblotting with K48- and K63-linkage-specific ubiquitin antibodies. Linkage-specific ubiquitination signals were normalized to the amount of immunoprecipitated RACGAP1.

To characterize the specific ubiquitin linkage and modification sites on RACGAP1, GC cells were first infected with UBE2S-lentivirus (oeUBE2S) or an empty vector control to establish stable cell lines. For the analysis of endogenous ubiquitin linkage, control and oeUBE2S GC cells were treated with 5 μM MG132 (MCE, Cat No. HY-13259) for 6 h prior to harvesting to block proteasomal degradation. Total protein was extracted and subjected to IP using an anti-RACGAP1 antibody. The specific linkage types were subsequently detected via immunoblotting using linkage-specific antibodies against K63-linked ubiquitin (Abcam, ab179434) or K48-linked ubiquitin (Abcam, ab140601).

For the K63-specific site-verification assay, GC cells were transiently co-transfected with Flag-tagged wild-type (WT) or mutant (K122R, K292R, K423R, K490R, K587R) RACGAP1 plasmids, together with HA-tagged K63-only ubiquitin (HA-K63-only-Ub) plasmids. Transfections were performed using Lipofectamine 3000 (Thermo Fisher Scientific, USA; Cat No. L3000075) according to the manufacturer’s instructions. All lentiviral vectors and plasmids were designed and synthesized by Genomeditech Biotechnology (Shanghai, China). After 48 h of transfection, cells were lysed under denaturing conditions, and RACGAP1 was immunoprecipitated using anti-Flag or anti-RACGAP1 antibodies. The polyubiquitinated RACGAP1 smears were subsequently visualized by immunoblotting with an anti-HA antibody to evaluate the site-specific K63-linked ubiquitination levels.

### 2.18. Site-directed mutagenesis and site prediction

Potential lysine (K) ubiquitination sites on the RACGAP1 protein were predicted using the PhosphoSitePlus database (https://www.phosphosite.org/). Based on these predictions, a series of lysine-to-arginine (K-R) mutants (K122R, K292R, K423R, K490R, and K587R) were constructed by Genomeditech (Shanghai, China).

### 2.19. Functional comparison of RACGAP1-WT and RACGAP1-K292R

To evaluate the functional importance of RACGAP1 K292, AGS cells stably expressing UBE2S or the corresponding vector control were transiently transfected with equal amounts of empty vector, *N*-terminal 3 × Flag-tagged RACGAP1-WT, or RACGAP1-K292R plasmids under identical transfection conditions. UBE2S-depleted AGS cells were also transfected with RACGAP1-WT or RACGAP1-K292R to confirm that both constructs could be expressed under low-UBE2S conditions. For proteasome-blockade experiments, cells were treated with 5 μM MG132 for 6 h before harvesting. At 48 h after transfection, whole-cell lysates were subjected to Western blotting for UBE2S, Flag, total JNK, *p*-JNK, total c-Jun, *p*-c-Jun, and the indicated loading control. For functional evaluation, cell viability and clonogenic growth were assessed using CCK-8 and colony formation assays, respectively, as described above. Cell migration was assessed using a wound-healing assay as previously described.

### 2.20. Pharmacological JNK inhibition and pathway-involvement assay

SP600125 (10 μM; TargetMol, Cat No. T3109) was used to inhibit JNK signaling. For immunoblotting, cells were treated with SP600125 or an equivalent volume of DMSO for 24 h before harvesting. For colony formation assays, cells were cultured in the continuous presence of 10 μM SP600125 or DMSO. The culture medium containing freshly diluted SP600125 or vehicle was replaced every 2–3 days. Whole-cell lysates were analyzed by Western blotting for UBE2S, RACGAP1, total JNK, *p*-JNK, total c-Jun, *p*-c-Jun, and the indicated loading control. Under the same experimental conditions, long-term clonogenic capacity was evaluated using colony formation assays.

### 2.21. Statistical analysis

Statistical analyzes were performed using SPSS Statistics version 25.0 (IBM Corp., Armonk, NY, USA), and data visualization was conducted using GraphPad Prism 9.0. Quantitative data are presented as the mean ± standard deviation (SD) from at least three independent biological replicates unless otherwise indicated. The exact sample size for each experiment is provided in the corresponding figure legends.

Data normality was assessed using the Shapiro-Wilk test, and homogeneity of variance was evaluated using Levene’s test. For comparisons between two groups, the unpaired Student’s t-test was used when the data were normally distributed and showed homogeneous variance; otherwise, the Mann-Whitney U test was applied. For comparisons among multiple groups, one-way analysis of variance (ANOVA) was performed, followed by the Least Significant Difference (LSD) post-hoc test when variances were equal or Tamhane’s T2 test when variances were unequal. Longitudinal tumor-volume data were analyzed using two-way repeated-measures ANOVA with treatment group and time as factors. Greenhouse–Geisser correction was applied when the sphericity assumption was violated, and Bonferroni correction was used for prespecified pairwise comparisons. Terminal tumor weights were analyzed separately using one-way ANOVA. All statistical tests were two-sided. A *P*-value < 0.05 was considered statistically significant (*P < 0.05, **P < 0.01, ***P < 0.001).

### 2.22. Reagent and antibody information

Detailed information on the suppliers, catalog numbers, and working concentrations or dilutions of the key reagents and antibodies used in this study is provided in Supplementary Table S2.

## 3. Results

### 3.1. UBE2S is upregulated in GC and shows exploratory associations with survival

To initiate our investigation, we performed a pan-cancer expression profiling of UBE2S across multiple malignancies using integrated TCGA and GTEx datasets. The results revealed a pervasive upregulation of UBE2S in a wide spectrum of tumor tissues compared to normal controls, with a notably prominent elevation observed in GC (Figure 1A, B). This observation was further corroborated by paired tissue analysis, which demonstrated significantly higher UBE2S transcript levels in GC lesions than in their adjacent normal counterparts (Figure 1C). An exploratory ROC analysis yielded an AUC of 0.909, indicating within-dataset discrimination between GC and normal samples in the analyzed public dataset (Figure 1D). This analysis was hypothesis-generating and should not be interpreted as evidence of clinically validated diagnostic performance because no independent external validation cohort was included.

**Figure 1.**
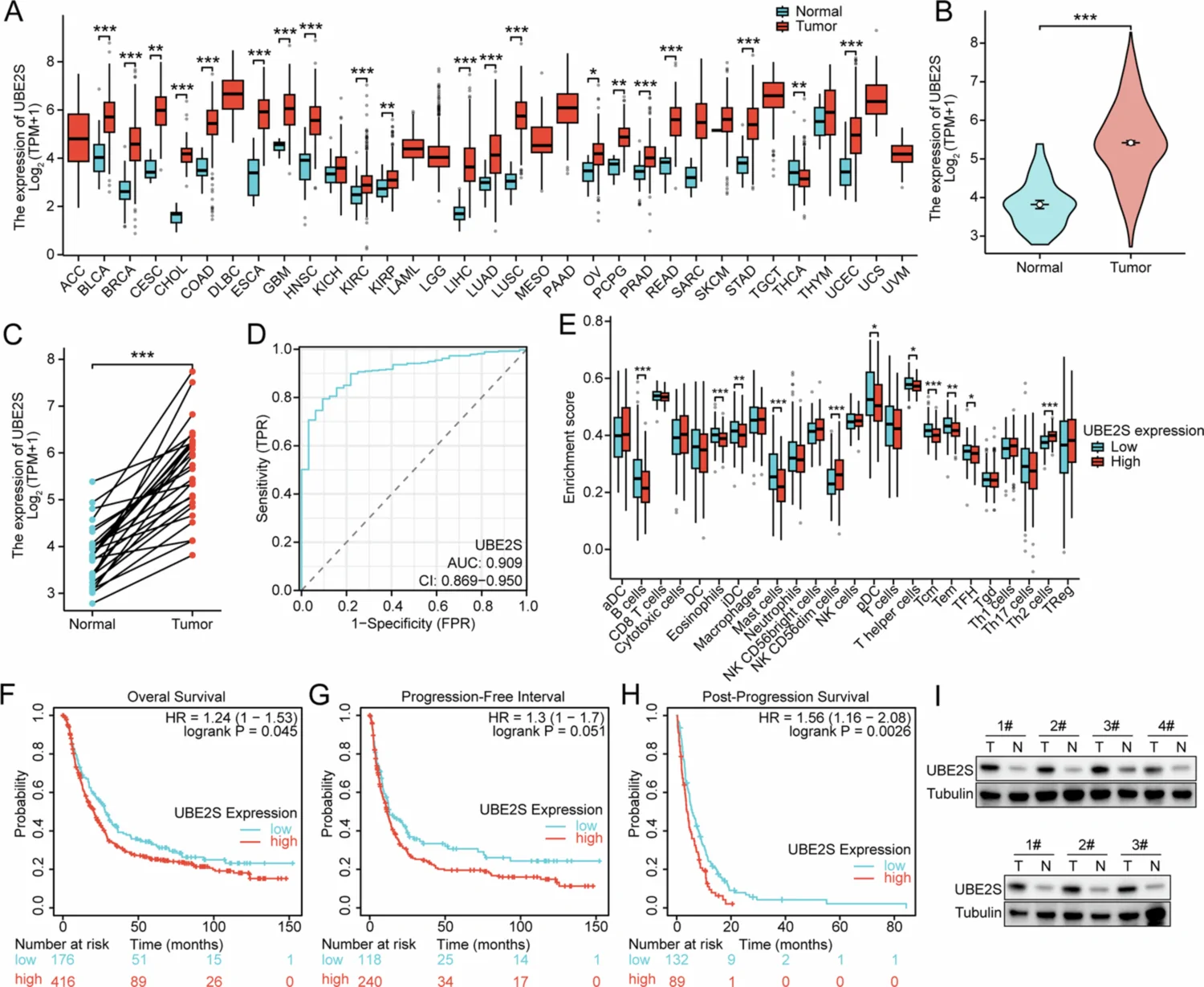
Elevated UBE2S expression in GC shows exploratory associations with survival and estimated immune-cell infiltration patterns. (A−B) Pan-cancer profiling of UBE2S mRNA levels across multiple human malignancies and a comparative analysis specifically focusing on GC versus normal gastric tissues. (C) Differential expression of UBE2S in paired GC lesions and adjacent non-tumor specimens. (D) Exploratory ROC analysis evaluating the ability of UBE2S expression to distinguish GC from normal samples in the analyzed public dataset. (E) Correlation between UBE2S expression and the abundance of diverse tumor-infiltrating immune cell (TIIC) subtypes. (F−H) Exploratory Kaplan–Meier plots generated using the auto-selected cutoff, illustrating the associations between UBE2S expression and Overall Survival (OS), Progression-Free Interval (PFI), and Post-Progression Survival (PPS) in GC cohorts. (Note: Panels A–E were based on public transcriptomic datasets, whereas panels F–H were generated using the Kaplan–Meier Plotter). (I) Western blot assessment of UBE2S protein expression in seven paired GC and adjacent non-tumor tissues.

Furthermore, we examined the association between UBE2S expression and survival outcomes in patients with GC. Exploratory Kaplan–Meier analysis using the auto-selected cutoff showed that high UBE2S expression was associated with shorter OS (*P* = 0.045) and PPS (*P* = 0.0026), whereas the association with PFI did not reach statistical significance (*P* = 0.051) (Figure 1F–H). As an exploratory bioinformatic observation, we also investigated the interplay between UBE2S and the tumor immune microenvironment using public datasets, identifying significant correlations with the estimated infiltration of specific immune cell subtypes, particularly Th2 cells and macrophages (Figure 1E). To preliminarily assess whether the expression pattern was also evident at the protein level, we analyzed seven paired fresh GC and adjacent non-tumor tissues from the Second Affiliated Hospital of Zhejiang Chinese Medical University. Consistent with our transcriptomic data, Western blot analysis showed higher UBE2S protein abundance in GC tissues than in the matched adjacent tissues within this small cohort (Figure 1I). Together, these findings indicate that UBE2S is upregulated in GC and shows an exploratory association with unfavorable survival under the auto-selected cutoff in public datasets, while the seven paired tissues provide preliminary protein-level support for its increased expression.

### 3.2. UBE2S silencing reduces GC cell viability, clonogenicity, and motility in vitro and impairs tumor growth in vivo

To elucidate the functional involvement of UBE2S in GC progression, we first characterized its expression profile across a panel of cell lines. Both Western blot and qRT-PCR analyzes demonstrated that UBE2S was markedly upregulated at both the protein and mRNA levels in various GC cell lines (including AGS, HGC-27, and MKN-1) relative to the immortalized normal gastric epithelial GES-1 cell line (Figure 2A,B). Subsequently, we established stable UBE2S-knockdown models in AGS and HGC-27 cells using two independent shRNAs, with the knockdown efficiency validated via immunoblotting (Figure 2C).

**Figure 2.**
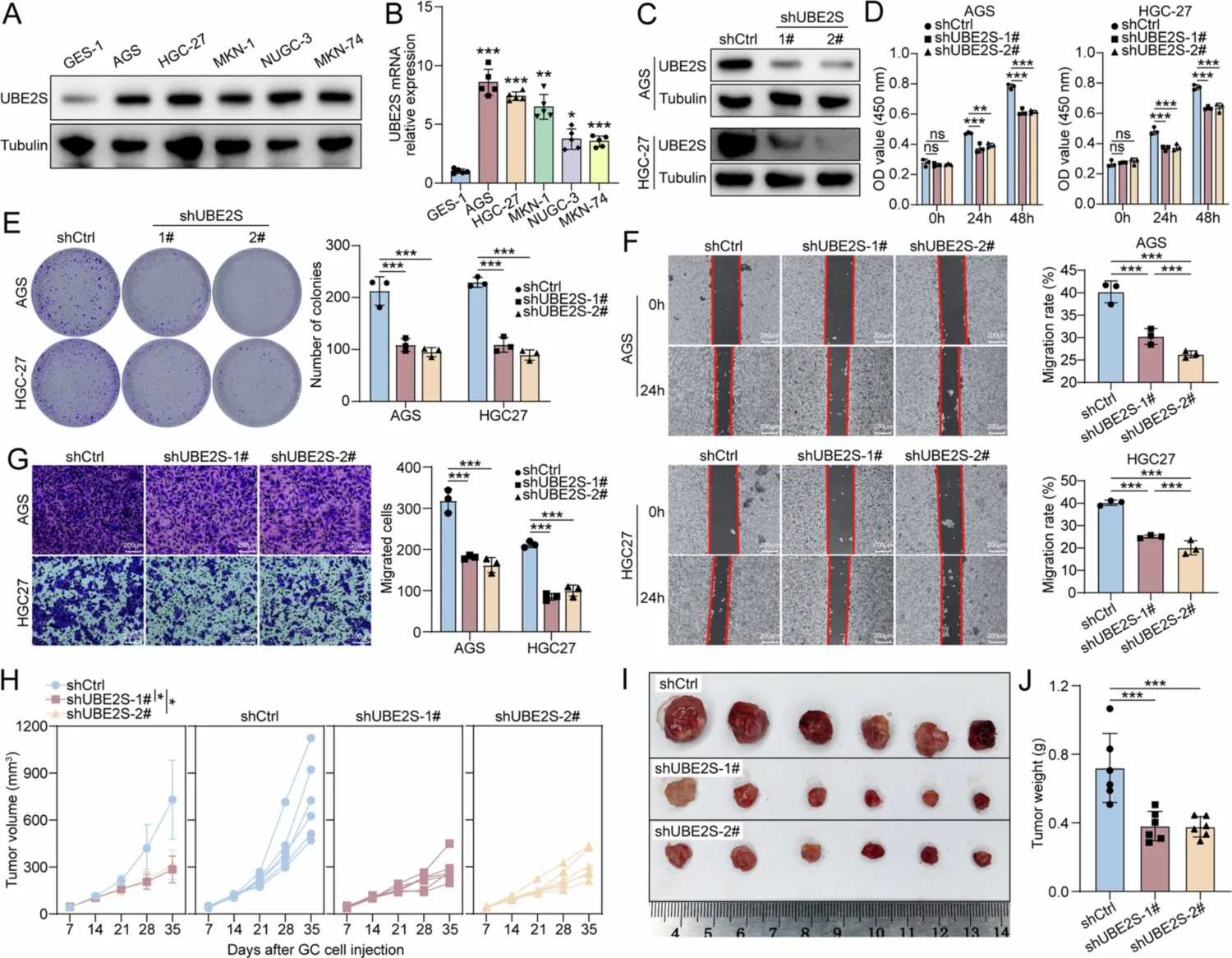
UBE2S silencing attenuates the malignant phenotypes of GC cells in vitro and suppresses xenograft tumor growth in vivo. (A−B) Assessment of baseline UBE2S protein and mRNA expression levels across a panel of human GC cell lines (AGS, HGC-27, MKN-1, NUGC-3, and MKN-74) and the immortalized gastric epithelial cell line GES-1, determined by Western blot and qRT-PCR analysis, respectively. (C) Immunoblotting validation of UBE2S knockdown efficiency in AGS and HGC-27 cells following transduction with two independent shRNAs (shUBE2S-1# and shUBE2S-2#) compared to the non-targeting control (shCtrl). (D) Cell viability assessment via CCK-8 assays, showing the OD values at 450 nm for shCtrl and shUBE2S groups at indicated time points (0, 24, and 48 h) (*n* = 3 per group). (E) Representative images and quantitative analysis of colony formation assays reflecting clonogenic capacity (*n* = 3 per group). (F) Micrographs of wound healing assays captured at 0 and 24 h post-scratching to evaluate lateral migration (*n* = 3 per group). (G) Representative images and statistical quantification of Transwell migration assays (*n* = 3 per group). (H−J) In vivo evaluation of UBE2S knockdown using an HGC-27 xenograft model (*n* = 6 per group), including subcutaneous tumor growth kinetics (H), macroscopic photographs of harvested tumors at the experimental endpoint (I), and terminal tumor weight measurements (J). Longitudinal tumor-volume data in (H) were analyzed using two-way repeated-measures ANOVA with Greenhouse–Geisser correction and Bonferroni-adjusted prespecified pairwise comparisons (shCtrl vs shUBE2S-1# and shCtrl vs shUBE2S-2#). Quantitative in vitro data and terminal tumor weights were analyzed by one-way ANOVA followed by LSD or Tamhane’s T2 post-hoc test, as appropriate. Data are presented as the mean ± SD. Exact pairwise comparisons are indicated in the corresponding panels.

Functional investigations revealed that UBE2S silencing significantly reduced GC cell viability, as measured by CCK-8 assays (Figure 2D). Consistent with these findings, colony formation assays showed that UBE2S depletion severely impaired the clonogenic capacity of both cell lines (Figure 2E). To evaluate the impact of UBE2S on *in vitro* cellular motility and migration, we performed wound healing and Transwell assays, which indicated that the migratory capability of GC cells was profoundly suppressed following UBE2S knockdown (Figure 2F,G).

To extend these observations in vivo, we established a subcutaneous xenograft model using HGC-27 cells. UBE2S depletion significantly altered tumor-growth trajectories over time, as demonstrated by a significant group × time interaction (*P* < 0.001). Prespecified pairwise comparisons further showed significant differences in growth trajectories between shCtrl and shUBE2S-1# (adjusted *P* = 0.0042) and between shCtrl and shUBE2S-2# (adjusted *P* = 0.0080) (Figure 2H). Upon termination of the experiment, a marked reduction in both tumor volume and final weight was observed in the UBE2S-depleted group (Figure 2I,J). Collectively, these data indicate that UBE2S contributes to the maintenance of GC cell viability, clonogenicity, migration, and xenograft tumor growth.

### 3.3. Proteomic profiling implicates UBE2S in cell-cycle-associated regulation

To decipher the molecular mechanisms through which UBE2S exerts its oncogenic effects in GC, we performed 4D-DIA quantitative proteomic analysis comparing shCtrl and shUBE2S-transfected HGC-27 cells. Principal component analysis (PCA) revealed that the protein expression profiles of the two groups were distinctly segregated, reflecting a substantial shift in the proteomic landscape upon UBE2S silencing (Figure 3A). Using the originally applied exploratory criteria of a fold change >1.5 or <0.67 together with a nominal Student’s t-test *P* < 0.05, we identified 397 candidate differentially abundant proteins, including 371 showing decreased abundance and 26 showing increased abundance following UBE2S depletion. Specifically, proteins including RACGAP1, NGAL, DNMT1, and PQBP1 showed decreased abundance, whereas proteins including NBEAL2, GC, CAMLG, and GDAP1 showed increased abundance (Figure 3B). Subcellular localization analysis indicated that the majority of these candidate proteins were predominantly partitioned within the nucleus and cytoplasm (Figure 3C). To further characterize the functional implications of these candidate proteins, we conducted KEGG and GO enrichment analyzes. KEGG pathway analysis highlighted that the candidate protein set showed enrichment in pathways including the cell cycle, cellular senescence, p53 signaling pathway, ECM-receptor interaction, and JAK-STAT signaling pathway (Figure 3D). Consistently, GSEA-KEGG analysis demonstrated a significant negative enrichment of the cell-cycle gene set following UBE2S knockdown (ES = −0.5941, FDR = 0.03501) (Figure 3E). Functional annotation via GO analysis revealed that these candidate proteins were intricately associated with functional categories including transcription regulator activity, molecular function regulator, and ATP-dependent activity (Figure 3F). Furthermore, GSEA-GO analysis showed negative enrichment of gene sets related to cell-cycle phase transition, cell-cycle processes, and cell division (Figure 3G–I). To experimentally validate the link between UBE2S and cell cycle regulation, we assessed the expression of key cell cycle checkpoints via Western blot. Consistent with these pathway-level findings, the protein levels of Cyclin D1 and CDK6 were dramatically diminished following UBE2S knockdown (Figure 3J). Taken together, these results demonstrate that UBE2S depletion suppresses cell-cycle-associated molecular programs and reduces Cyclin D1 and CDK6 expression, consistent with reduced cell-cycle-associated activity.

**Figure 3.**
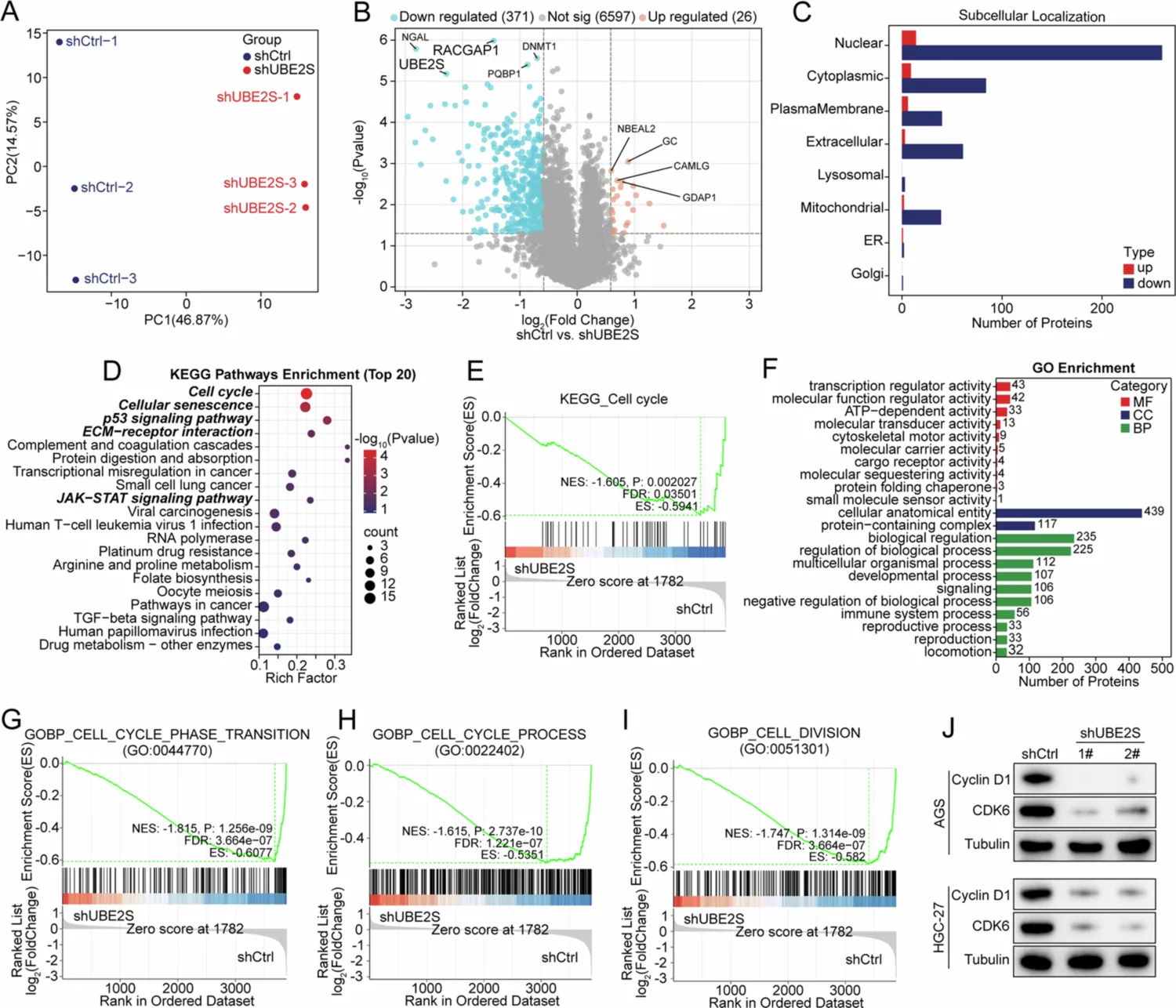
Quantitative proteomics reveals suppression of cell-cycle-associated programs following UBE2S depletion. (A) Principal component analysis (PCA) comparing the protein expression profiles of HGC-27 GC cells transfected with either shCtrl or shUBE2S. The 4D-DIA proteomic analysis included three independent biological replicates per group (*n* = 3). (B) Volcano plot displaying candidate differentially abundant proteins identified using the exploratory criteria of a fold change >1.5 or <0.67 together with a nominal Student’s t-test *P* < 0.05 following UBE2S silencing. (C) Distribution of the candidate differentially abundant proteins across various subcellular compartments. (D) KEGG pathway enrichment analysis of the candidate protein set. (E) GSEA-KEGG showing negative enrichment of the cell-cycle gene set following UBE2S depletion. (F) Gene Ontology (GO) functional annotation of the candidate protein set, categorized by biological process (BP), molecular function (MF), and cellular component (CC). (G–I) GSEA plots demonstrating significant negative enrichment of gene sets related to (G) cell cycle phase transition, (H) cell cycle process, and (I) cell division in UBE2S-silenced cells. (J) Western blot analysis validating the expression of key cell cycle regulators (Cyclin D1 and CDK6) in shCtrl and UBE2S-depleted HGC-27 cells.

### 3.4. UBE2S positively regulates RACGAP1 and activates the JNK/c-Jun signaling axis

Previous studies have characterized RACGAP1 as a pivotal proto-oncogene that orchestrates malignant progression by modulating the JNK/c-Jun/AP-1 signaling cascade, a conserved axis governing cell cycle dynamics and survival across multiple cancers.^28^ Given that RACGAP1 emerged as a prominently downregulated candidate in our proteomic screen upon UBE2S silencing, we sought to determine whether UBE2S exploits the RACGAP1-dependent JNK pathway to drive GC progression. Bioinformatic interrogation of TCGA and GTEx datasets showed higher RACGAP1 expression in GC tissues than in normal tissues (Figure 4A,B), and exploratory Kaplan–Meier analysis using the auto-selected cutoff showed an association between elevated RACGAP1 expression and unfavorable survival (Figure 4C). Crucially, a positive correlation was observed between the transcript levels of UBE2S and RACGAP1 in GC cohorts (Spearman R = 0.573, *P* < 0.001) (Figure 4D). To validate this regulatory link at the protein level, Western blot analysis was performed. We observed that UBE2S knockdown markedly diminished RACGAP1 protein abundance. Building on the known capacity of RACGAP1 to activate the JNK phosphorylation cascade, we further examined the status of this pathway. Notably, the reduction in RACGAP1 was accompanied by decreased JNK and c-Jun phosphorylation, as reflected by lower *p*-JNK/total JNK and *p*-c-Jun/total c-Jun ratios (Figure 4E). To verify whether UBE2S-mediated activation of this signaling axis is functionally contingent upon RACGAP1, we performed a genetic rescue experiment. Ectopic expression of RACGAP1 (oeRACGAP1) in UBE2S-knockdown cells partially restored the *p*-JNK/total JNK and *p*-c-Jun/total c-Jun ratios (Figure 4F−G). These results indicate that RACGAP1 contributes to UBE2S-dependent activation of the JNK/c-Jun signaling pathway in GC cells.

**Figure 4.**
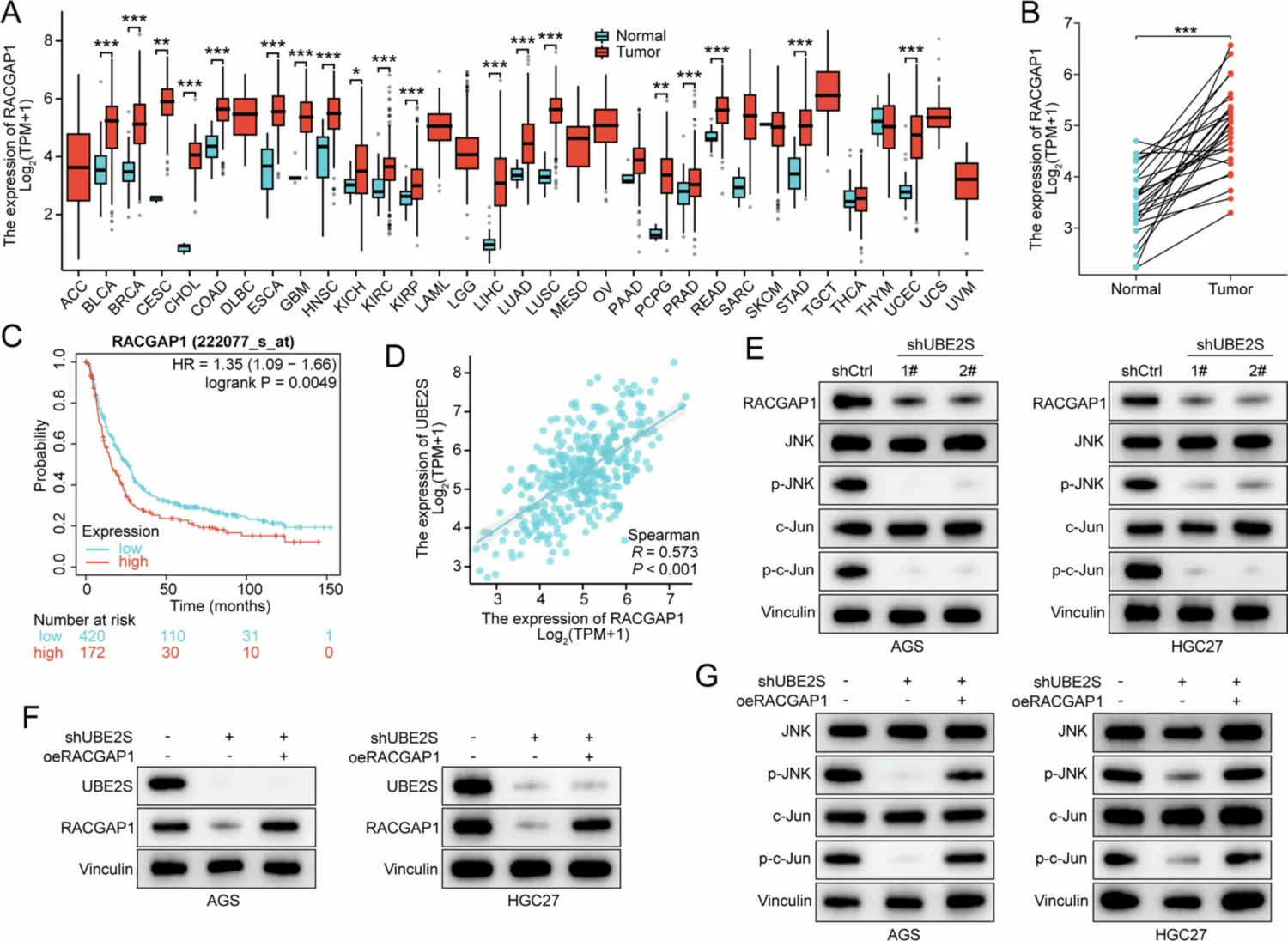
UBE2S maintains RACGAP1 protein abundance and supports JNK/c-Jun activation in GC cells. (A−B) Pan-cancer expression profiling of RACGAP1 across 33 TCGA malignancies and a comparative analysis of RACGAP1 levels in paired GC and adjacent non-tumor tissues. (C) Exploratory Kaplan–Meier survival analysis using the auto-selected cutoff, illustrating the association between RACGAP1 expression and overall survival in GC patients. (D) Spearman's rank correlation analysis demonstrating a significant positive association between UBE2S and RACGAP1 mRNA expression in the TCGA-STAD cohort (R = 0.573, *P* < 0.001). (E) Western blot detection of RACGAP1, total JNK, *p*-JNK, total c-Jun, and *p*-c-Jun protein levels in GC cells (AGS and HGC-27) following stable knockdown of UBE2S. (F−G) Western blot detection of RACGAP1, total JNK, *p*-JNK, total c-Jun, and *p*-c-Jun in the rescue experiments.

### 3.5. Restoration of RACGAP1 rescues the inhibitory effects of UBE2S depletion on GC progression

To determine whether RACGAP1 functions as a major downstream mediator of UBE2S, we performed a series of genetic rescue experiments. CCK-8 assays showed that the reduction in cell viability caused by UBE2S silencing was significantly reversed by ectopic RACGAP1 expression (Figure 5A). Consistently, colony formation assays demonstrated that RACGAP1 restoration rescued the impaired clonogenic capacity of UBE2S-depleted cells (Figure 5B). Wound-healing and Transwell assays further showed that RACGAP1 re-expression significantly restored the cellular motility suppressed by UBE2S knockdown (Figure 5C,D). To further assess pathway dependency, we performed pharmacological JNK inhibition using SP600125 in UBE2S-depleted AGS cells. RACGAP1 re-expression restored *p*-JNK/total JNK and *p*-c-Jun/total c-Jun levels, whereas SP600125 treatment reduced the restored *p*-JNK/JNK and *p*-c-Jun/c-Jun levels without affecting RACGAP1 abundance, consistent with JNK/c-Jun signaling acting downstream of RACGAP1 (Figure 5E). Consistently, RACGAP1 re-expression rescued colony formation after UBE2S knockdown, while JNK inhibition largely abolished this effect and reduced colony numbers to levels comparable with the shUBE2S group (Figure 5F). These results support the involvement of JNK/c-Jun signaling, at least in part, in RACGAP1-mediated clonogenic rescue.

**Figure 5.**
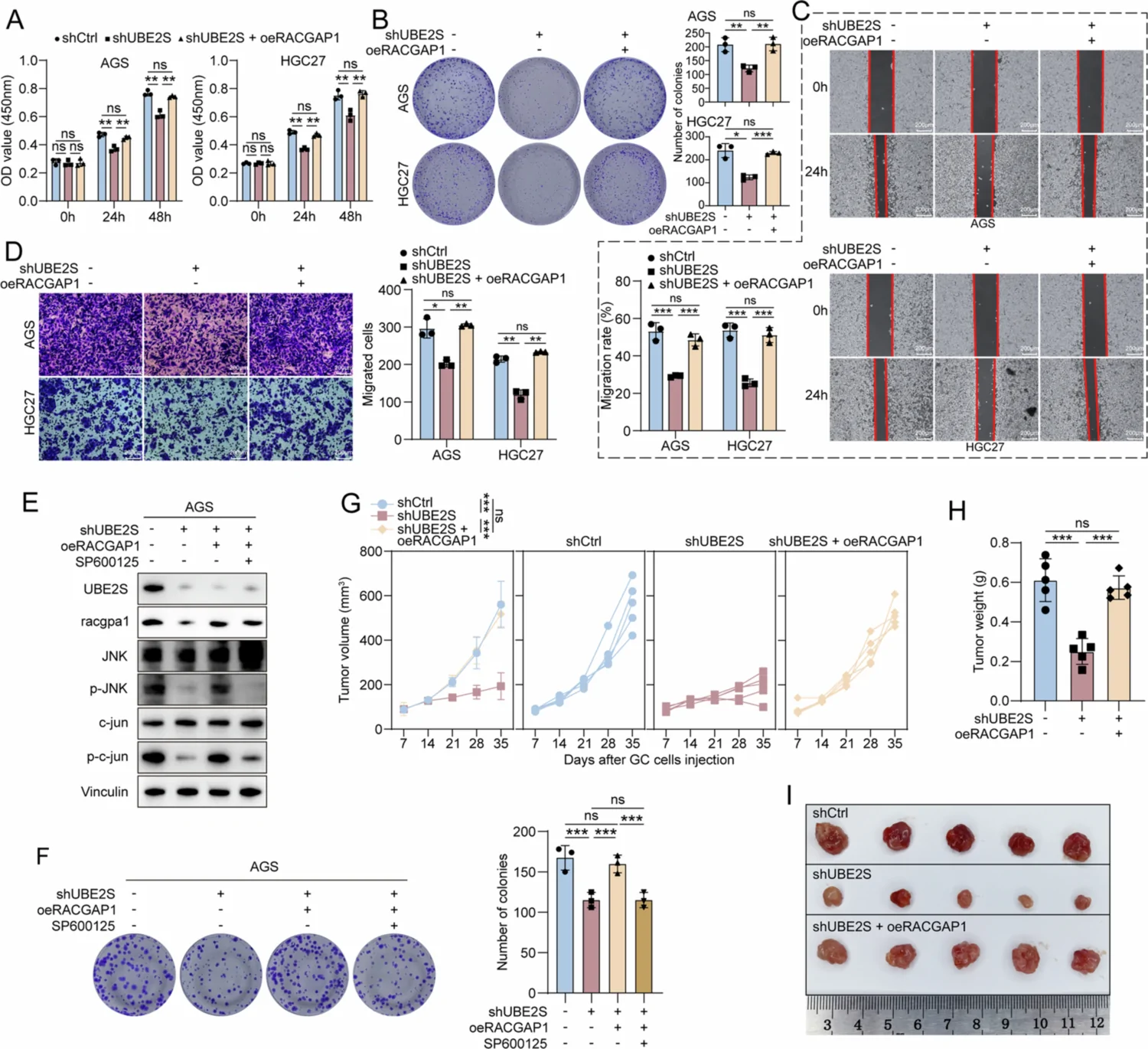
RACGAP1 restoration effectively rescues the malignant phenotypes suppressed by UBE2S knockdown. (A–B) Assessment of cell viability and clonogenic capacity in GC cells (AGS and HGC-27) transfected with shCtrl, shUBE2S, or shUBE2S + oe-RACGAP1, as determined by CCK-8 assays (A) and colony formation assays (B) (*n* = 3 per group). (C–D) Evaluation of cellular motility capacity through wound-healing assays (C) and Transwell migration assays (D) across the indicated transfection groups (*n* = 3 per group). (E) Western blot analysis of UBE2S, RACGAP1, total JNK, *p*-JNK, total c-Jun, and *p*-c-Jun in the indicated AGS groups with or without SP600125-mediated pharmacological JNK inhibition. (F) Representative images and quantification of colony formation under the same conditions (*n* = 3 per group). (G–I) *In vivo* validation of the functional rescue effect using an HGC-27 xenograft model (*n* = 5 per group). The analysis includes subcutaneous tumor growth kinetics (G), representative macroscopic photographs of harvested tumors (H), and statistical quantification of tumor weights (I) at the experimental endpoint (Day 35). Longitudinal tumor-volume data in (G) were analyzed using two-way repeated-measures ANOVA with Greenhouse–Geisser correction and Bonferroni-adjusted prespecified pairwise comparisons (shCtrl vs shUBE2S and shUBE2S vs shUBE2S + oeRACGAP1). Quantitative in vitro data and terminal tumor weights were analyzed by one-way ANOVA followed by LSD or Tamhane’s T2 post-hoc test, as appropriate. Data are presented as the mean ± SD. Exact pairwise comparisons are indicated in the corresponding panels.

To validate the functional relationship between UBE2S and RACGAP1 *in vivo*, we established a subcutaneous xenograft rescue model. Consistent with the *in vitro* findings, longitudinal analysis revealed a significant group × time interaction (*P* < 0.001). Prespecified pairwise comparisons further showed significant differences in tumor-growth trajectories between shCtrl and shUBE2S and between shUBE2S and shUBE2S + oeRACGAP1 (both adjusted *P* < 0.001), indicating that RACGAP1 overexpression substantially reversed the growth suppression caused by UBE2S depletion (Figure 5G). This rescue effect was further supported by harvested tumor appearance and terminal tumor weight (Figure 5H,I). Collectively, these results identify RACGAP1 as a major downstream mediator of UBE2S-dependent GC cell growth, motility, and xenograft tumor growth, and support a contribution of downstream JNK/c-Jun signaling to RACGAP1-mediated clonogenic rescue.

### 3.6. UBE2S-dependent stabilization of RACGAP1 is associated with K292-dependent K63-linked polyubiquitination

Finally, we investigated the post-translational mechanism by which UBE2S regulates RACGAP1 protein abundance. Although UBE2S knockdown markedly reduced RACGAP1 protein levels, it did not significantly alter RACGAP1 mRNA expression (Figure 6A), indicating that UBE2S regulates RACGAP1 predominantly at the post-translational level. Reciprocal Co-IP assays confirmed an association between endogenous UBE2S and RACGAP1 in GC cells (Figure 6B,C). A CHX chase assay further showed that UBE2S overexpression prolonged the half-life of RACGAP1 protein compared with the vector control (Figure 6D), supporting a role for UBE2S in maintaining RACGAP1 protein stability.

**Figure 6.**
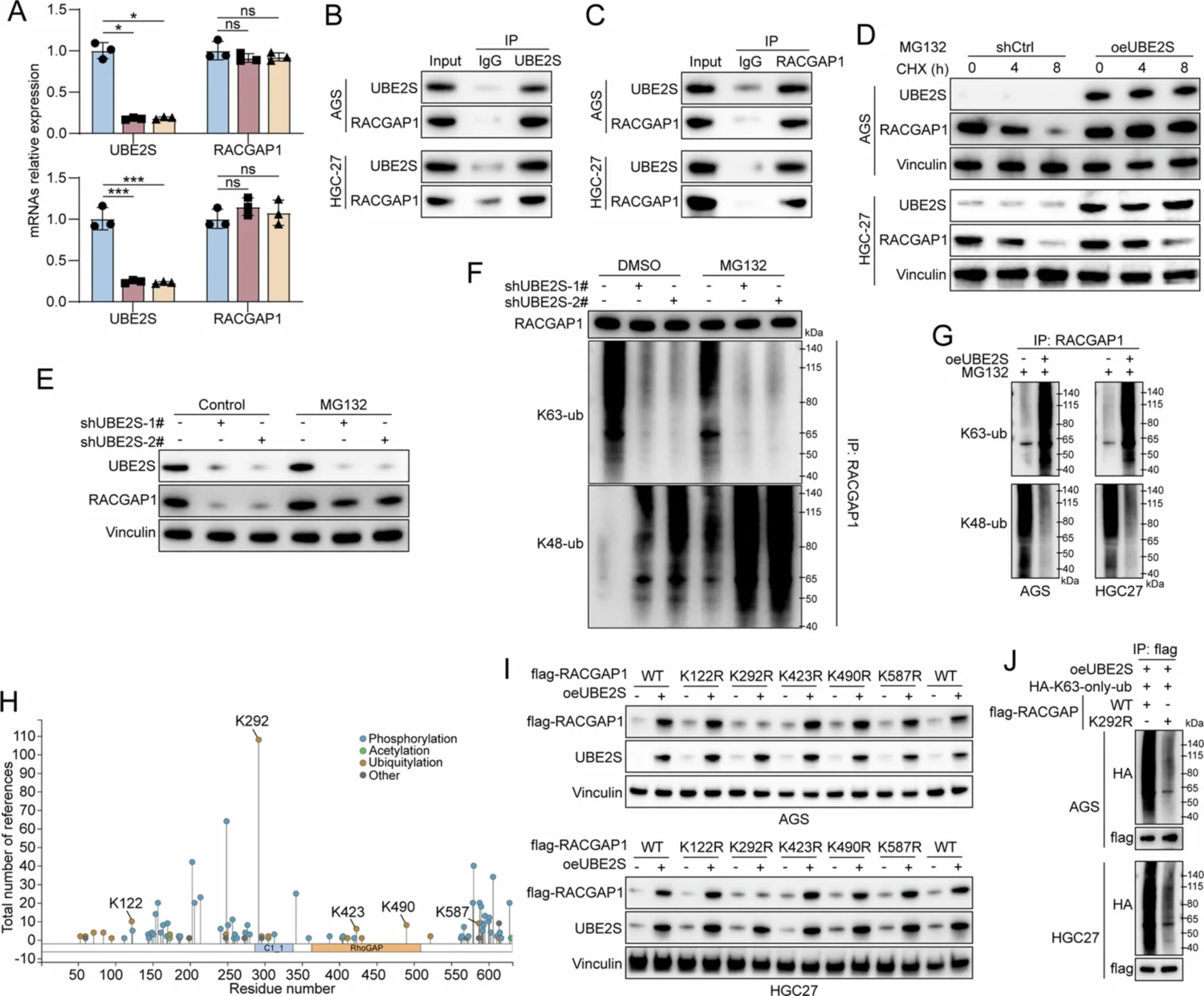
UBE2S-dependent RACGAP1 stabilization is associated with K292-dependent K63-linked ubiquitination and reciprocal changes in K48-linked ubiquitination. (A) qRT-PCR analysis of UBE2S and RACGAP1 mRNA expression in GC cells following stable knockdown of UBE2S (*n* = 3 per group). (B–C) Co-IP assays detecting the endogenous association between UBE2S and RACGAP1 in AGS and HGC-27 cells. (D) Protein stability analysis via CHX chase assays at indicated time points in cells overexpressing UBE2S compared to control cells. (E) Western blot analysis of UBE2S and RACGAP1 protein abundance in shCtrl-, shUBE2S-1#-, and shUBE2S-2#-expressing cells treated with vehicle or MG132. (F) Endogenous RACGAP1 was immunoprecipitated from the indicated cells, followed by immunoblotting with K48- and K63-linkage-specific ubiquitin antibodies. Linkage-specific ubiquitination signals were normalized to immunoprecipitated RACGAP1. (G) IP of RACGAP1 followed by immunoblotting with K63-linked and K48-linked ubiquitin-specific antibodies in control or UBE2S-overexpressing cells. (H) Schematic representation of potential lysine (K) ubiquitination sites on the RACGAP1 protein based on PhosphoSitePlus databases. (I) Western blot analysis comparing the protein levels of Flag-tagged wild-type (WT) RACGAP1 and various lysine-to-arginine (K-R) mutants (K122R, K292R, K423R, K490R, K587R) in the presence or absence of UBE2S overexpression. (J) Ubiquitination assay utilizing a K63-only ubiquitin mutant (HA-K63-only-Ub) to detect the K63-linked polyubiquitination levels of RACGAP1-WT versus the K292R mutant under UBE2S overexpression conditions. For panel A, data are presented as the mean ± SD and were analyzed by one-way ANOVA followed by the appropriate post-hoc test.

To obtain reciprocal loss-of-function evidence, we next examined RACGAP1 protein abundance and ubiquitination following UBE2S depletion. Under vehicle-treated conditions, two independent UBE2S-targeting shRNAs markedly reduced RACGAP1 protein levels. Treatment with the proteasome inhibitor MG132 partially restored RACGAP1 abundance in UBE2S-depleted cells without reversing UBE2S knockdown (Figure 6E), indicating that the reduction in RACGAP1 caused by UBE2S depletion is mediated, at least in part, by proteasome-dependent degradation. We subsequently immunoprecipitated endogenous RACGAP1 and examined its linkage-specific ubiquitination. After normalization to the amount of immunoprecipitated RACGAP1, UBE2S depletion markedly decreased K63-linked ubiquitination while increasing K48-linked ubiquitination of RACGAP1. MG132 treatment further increased the accumulation of K48-linked ubiquitinated RACGAP1 in UBE2S-depleted cells but did not restore K63-linked ubiquitination (Figure 6F). Conversely, UBE2S overexpression enhanced K63-linked ubiquitination and reduced K48-linked ubiquitination of RACGAP1 (Figure 6G). These reciprocal gain- and loss-of-function results support an association between UBE2S expression, increased K63-linked ubiquitination, reduced K48-linked ubiquitination, and enhanced RACGAP1 stability.

To identify the lysine residue required for UBE2S-dependent RACGAP1 stabilization, five candidate lysine residues—K122, K292, K423, K490, and K587—were selected based on the PhosphoSitePlus database (Figure 6H). UBE2S overexpression increased the protein abundance of RACGAP1-WT and the K122R, K423R, K490R, and K587R mutants but failed to comparably stabilize the K292R mutant (Figure 6I). Moreover, in cells expressing HA-tagged K63-only ubiquitin, the K292R mutation markedly reduced the UBE2S-associated increase in K63-linked ubiquitination of RACGAP1 (Figure 6J). These findings indicate that K292 is required for the full UBE2S-associated increase in K63-linked ubiquitination and stabilization of RACGAP1.

### 3.7. RACGAP1 K292 is required for full UBE2S-dependent JNK/c-Jun activation and malignant phenotypes in vitro

To determine whether K292 is functionally required for the biological effects associated with UBE2S-mediated RACGAP1 stabilization, we directly compared Flag-tagged RACGAP1-WT and RACGAP1-K292R. Both constructs were readily expressed at comparable levels in UBE2S-depleted AGS cells, indicating that the K292R construct was not intrinsically defective in expression (Figure 7A).

**Figure 7.**
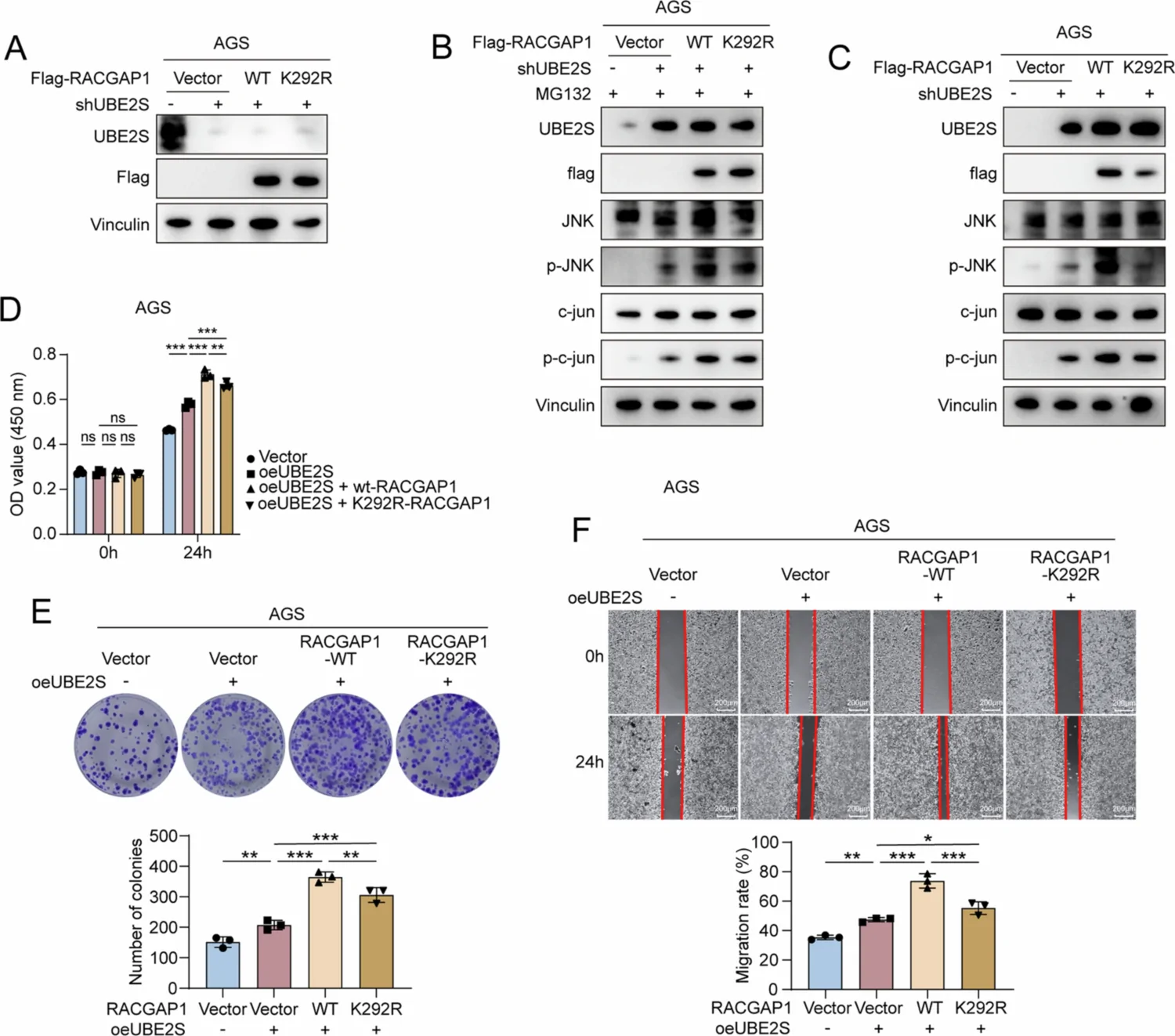
RACGAP1 K292 is required for full UBE2S-dependent stabilization, JNK/c-Jun activation, cell viability, clonogenic growth, and migration in vitro. (A) Western blot analysis confirming the expression of Flag-tagged RACGAP1-WT and RACGAP1-K292R in UBE2S-depleted AGS cells. (B) Western blot analysis of UBE2S, Flag-RACGAP1, total JNK, *p*-JNK, total c-Jun, and *p*-c-Jun in vector control, UBE2S-overexpressing, UBE2S-overexpressing plus RACGAP1-WT, and UBE2S-overexpressing plus RACGAP1-K292R cells following MG132 treatment. (C) Western blot analysis of UBE2S, Flag-RACGAP1, total JNK, *p*-JNK, total c-Jun, and *p*-c-Jun under the indicated conditions in the absence of MG132. (D) CCK-8 analysis comparing cell viability among vector control, oeUBE2S, oeUBE2S + RACGAP1-WT, and oeUBE2S + RACGAP1-K292R AGS cells (*n* = 3 per group). (E) Representative images and quantitative analysis of colony formation assays in the indicated groups (*n* = 3 per group). (F) Representative images and quantitative analysis of wound-healing assays in the indicated groups (*n* = 3 per group). Images were acquired at 0 and 24 h, and the wound-closure rate was calculated relative to the initial wound area or width. Data are presented as the mean ± SD. Statistical comparisons in (D–F) were performed using one-way ANOVA followed by LSD or Tamhane’s T2 post-hoc test, as appropriate. Exact pairwise comparisons are indicated in the corresponding panels.

We next compared WT and K292R under identical UBE2S-overexpressing conditions. Following MG132 treatment, the difference in Flag-RACGAP1 abundance between WT and K292R was largely diminished. Under these conditions, the *p*-JNK/total JNK and *p*-c-Jun/total c-Jun levels were also comparable between the WT and K292R groups (Figure 7B). In contrast, in the absence of MG132, RACGAP1-WT accumulated to a substantially higher level than RACGAP1-K292R in response to UBE2S overexpression. Consistent with this difference in protein abundance, the K292R group exhibited significantly lower *p*-JNK/total JNK and *p*-c-Jun/total c-Jun ratios than the WT group, whereas total JNK and total c-Jun levels remained largely unchanged (Figure 7C).

Functional assays yielded concordant results. UBE2S overexpression increased cell viability and clonogenic growth, and these effects were further enhanced by RACGAP1-WT. Notably, RACGAP1-K292R remained functionally active: compared with UBE2S overexpression alone, expression of the K292R mutant significantly increased cell viability and colony formation. Nevertheless, these effects were significantly weaker than those induced by RACGAP1-WT and occurred in parallel with the lower steady-state abundance of the K292R protein (Figure 7D,E). A similar pattern was observed in the wound-healing assay. RACGAP1-K292R significantly increased wound closure compared with UBE2S overexpression alone, confirming that the mutant retained promigratory activity. However, its effect was significantly weaker than that of RACGAP1-WT, consistent with the reduced accumulation of K292R-RACGAP1 in the absence of proteasome inhibition (Figure 7F).

Collectively, these findings indicate that RACGAP1-K292R remains biologically competent but cannot accumulate to the same extent as RACGAP1-WT under UBE2S-overexpressing conditions. The resulting reduction in protein abundance is accompanied by attenuated JNK/c-Jun activation, cell viability, clonogenic growth, and migration. Thus, K292 appears to regulate the magnitude of RACGAP1-mediated oncogenic output primarily by enabling UBE2S-dependent protein stabilization, rather than by determining the intrinsic functional activity of RACGAP1.

## 4. Discussion

The ubiquitin-conjugating enzyme (E2) family serves as a sophisticated regulatory stratum that fine-tunes oncogenic signaling by orchestrating specific ubiquitin chain topologies.^20^ As a critical Class I E2 enzyme, UBE2S has garnered significant attention for its multifaceted roles in driving cancer hallmarks, including chromosomal instability and therapy resistance.^29^ While its canonical function centers on APC/C-dependent K11-linked polyubiquitination during cell cycle progression,^30^ recent evidence has expanded its functional repertoire to include the modulation of metastasis-related substrates like *β*-catenin and LPP.^31^ Despite these insights in various solid tumors, the pathological significance of UBE2S in GC has remained underexplored. In this study, UBE2S was upregulated in GC, and higher expression showed an exploratory association with unfavorable survival in public datasets when patients were stratified using the auto-selected cutoff. Functional experiments further showed that UBE2S depletion reduced GC cell viability, clonogenicity, migration, and xenograft tumor growth. These findings support a tumor-promoting role for UBE2S in GC and provide a basis for further mechanistic and preclinical investigation of UBE2S in GC. However, these findings do not establish UBE2S as a clinically validated diagnostic, prognostic, or therapeutic biomarker, and its clinical relevance requires validation in larger independent cohorts.

To delineate the molecular networks regulated by UBE2S in GC, we employed unbiased quantitative proteomics, which prioritized RACGAP1 as a candidate downstream effector whose protein abundance was markedly reduced upon UBE2S knockdown. Originally recognized as a Rho-GTPase-activating protein (GAP) indispensable for cytokinesis and central spindle assembly, RACGAP1 has recently emerged as an oncogenic factor across various human malignancies.^32^ In colorectal cancer, for instance, RACGAP1 is frequently overexpressed, and its depletion precipitates G2/M phase arrest and apoptosis, underscoring its necessity for malignant cell survival.^33^ Expanding beyond its canonical role in cell division, RACGAP1 also functions as a versatile signaling scaffold and molecular chaperone. In prostate cancer, nuclear RACGAP1 participates in a reciprocal positive feedback loop with the androgen receptor (AR); specifically, it stabilizes both AR and its variant AR-V7 by impeding their interaction with the E3 ligase MDM2, thereby conferring resistance to endocrine therapy.^34^ Similarly, in cervical cancer, RACGAP1 accelerates malignant progression by modulating the AP-1 signaling axis through the dual mechanism of miR-192 suppression and *p*-JNK activation.^28^ Our results showed that pharmacological JNK inhibition suppressed JNK and c-Jun phosphorylation without reducing restored RACGAP1 abundance, supporting the placement of JNK/c-Jun downstream of RACGAP1. JNK inhibition also significantly abrogated the RACGAP1-mediated restoration of colony formation, reducing clonogenic growth to a level comparable to that observed after UBE2S depletion. These findings support a functional contribution of JNK/c-Jun signaling to the RACGAP1-mediated clonogenic phenotype. However, because SP600125 is a pharmacological inhibitor with recognized off-target effects, these data should not be interpreted as definitive genetic proof of JNK/c-Jun dependency. Genetic inhibition of JNK or c-Jun would provide stronger confirmation in future studies.

The functional fate of a substrate protein is fundamentally dictated by the “ubiquitin code,” wherein distinct polyubiquitin chain topologies—such as K11, K29, K48, and K63—elicit divergent cellular responses.^35^ While K48-linked polyubiquitination is the canonical signal for 26S proteasomal degradation, non-proteolytic linkages, particularly K63 chains, are increasingly recognized for their roles in protein stabilization, scaffolding, and signal transduction.^36^ An emerging paradigm in the ubiquitin field is the “competitive occupancy” or “steric hindrance” model, where non-degradative ubiquitin chains (e.g., K63) occupy specific lysine residues, thereby physically masking these sites from the attachment of degradative K48-linked chains. This reciprocal regulation has been observed in various oncogenic contexts. For instance, in glioma, TRIM26-mediated K63-linked ubiquitination stabilizes GPX4 to suppress ferroptosis, effectively counteracting its degradative turnover.^36^ In the present study, reciprocal gain- and loss-of-function experiments revealed an inverse association between K63- and K48-linked ubiquitination of RACGAP1. UBE2S overexpression increased K63-linked and reduced K48-linked ubiquitination, whereas UBE2S depletion produced the opposite pattern. Consistently, MG132 partially restored RACGAP1 protein abundance after UBE2S knockdown and promoted the accumulation of K48-linked ubiquitinated RACGAP1, supporting the involvement of proteasome-dependent turnover.

Mutational analysis further identified K292 as a key determinant of UBE2S-dependent RACGAP1 stabilization. Under UBE2S-overexpressing conditions, RACGAP1-K292R accumulated to a lower level than RACGAP1-WT and showed weaker JNK/c-Jun activation, cell viability, clonogenic growth, and wound closure. Proteasome inhibition largely reduced the differences in RACGAP1 abundance and downstream signaling between WT and K292R, whereas K292R retained partial biological activity in the absence of MG132. These findings suggest that K292 primarily regulates the magnitude of RACGAP1 signaling by enabling protein stabilization and accumulation rather than by determining its intrinsic functional competence. This interpretation is consistent with evidence that ubiquitin-chain topology and site-specific ubiquitination can differentially regulate protein stability, turnover, and signaling outputs.^8^ ^,^ ^37^ Nevertheless, our data do not establish that UBE2S directly assembles K63-linked chains on K292 or that K63-linked modification physically competes with K48-linked ubiquitination at the same residue. Cell-free ubiquitination assays and site-resolved ubiquitin proteomics will be required to resolve these mechanisms.

This study also has several limitations. Protein-level validation was restricted to seven paired GC specimens, and the ROC and survival analyzes lacked independent external validation; moreover, the survival analyzes relied on an auto-selected cutoff, which may introduce optimism and cutoff-selection bias.^38^ Accordingly, these clinical observations should be considered hypothesis-generating rather than evidence of validated diagnostic or prognostic utility. Although two independent shRNAs and rescue experiments reduce concerns regarding off-target effects, they cannot exclude them completely. Similarly, pathway dependency was assessed pharmacologically using SP600125, which has recognized off-target effects; therefore, genetic inhibition of JNK or c-Jun would provide more specific confirmation of this signaling relationship in future studies. In addition, subcutaneous xenografts in immunodeficient mice do not fully reproduce the anatomical context, metastatic behavior, or native tumor microenvironment of human GC, limiting the translational interpretation of the in vivo findings.^39^ More complex orthotopic and metastatic models can better preserve tissue context and relevant microenvironmental interactions. The immune-infiltration analysis should likewise be regarded as exploratory because it was not experimentally validated. Finally, although reciprocal UBE2S perturbation, MG132 rescue, and K292 mutagenesis support a K292-dependent K63-linked stabilization model, direct catalysis, site-specific ubiquitination, and K63/K48 competition remain unproven. Larger independent clinical cohorts, more physiologic in vivo models, genetic validation of JNK/c-Jun dependency, cell-free ubiquitination assays, and site-resolved mass spectrometry are therefore warranted.

## 5. Conclusion

In conclusion, our study identifies UBE2S as an oncogenic regulator of GC progression and RACGAP1 as a major downstream effector of UBE2S. Reciprocal gain- and loss-of-function experiments, proteasome inhibition, and K292 mutagenesis support a model in which UBE2S-dependent stabilization of RACGAP1 is associated with increased K63-linked and reduced K48-linked ubiquitination, with K292 being required for the full stabilization response. Importantly, the present experiments do not establish that UBE2S directly catalyzes K63-linked ubiquitination at K292 or that K63- and K48-linked ubiquitin chains directly compete for the same residue. RACGAP1-K292R retained partial biological activity, suggesting that K292 primarily modulates the magnitude of UBE2S-dependent RACGAP1 accumulation rather than determining the intrinsic biological activity of RACGAP1. Collectively, these findings support the functional relevance of the UBE2S/RACGAP1/JNK/c-Jun axis in GC, while any diagnostic, prognostic, or therapeutic implications remain hypothesis-generating and require independent clinical validation.

## Supplementary Material

Supplementary Table S1.docx Supplementary Table S1.docx

Supplementary Table S2.docx Supplementary Table S2.docx

Author Checklist.pdf Author Checklist.pdf

## Data Availability

The RNA-sequencing and clinical data analyzed in this study are publicly available from TCGA-STAD through the Genomic Data Commons portal and from the GTEx database. Survival analyzes were performed using the Kaplan-Meier Plotter database. The 4D-DIA proteomics data, processed expression matrices, numerical values underlying the graphs, uncropped Western blot images, qRT-PCR data, tumor volume and tumor weight measurements, and other datasets supporting the findings of this study have been deposited in Figshare and are openly available at https://doi.org/10.6084/m9.figshare.32669085. Any data that cannot be publicly shared due to ethical or privacy restrictions are available from the corresponding author upon reasonable request and with appropriate institutional approval.

## Protocol registration

The study protocol, including the research question, experimental design, and statistical analysis plan, was not preregistered in a publicly accessible repository. All experimental procedures were conducted in accordance with standard laboratory practices and established reporting guidelines.
