# Supplementary material for: UBE2S promotes gastric cancer progression through RACGAP1 stabilization associated with K292-dependent K63-linked ubiquitination
Source: Cancer Biol Ther. 2026 Oct 8;27(1):2737476. doi: 10.1080/15384047.2026.2737476 (PMC13652055; doi:10.1080/15384047.2026.2737476)
Supplement: Supplementary file 2 [file KCBT_A_2737476_SM7793.docx]

**Supplementary Table S2. Key reagents and antibodies used in this study**

**Part A.** Primary and secondary antibodies

| **Antibody** | **dilution** | **Supplier** | **Catalogue No.** |
| --- | --- | --- | --- |
| UBE2S | WB, 1:1,000; IP, 2 μg/IP | Proteintech | 14115-1-AP |
| RACGAP1 | WB, 1:1,000; IP, 2 μg/IP | Proteintech | 13739-1-AP |
| JNK | WB, 1:1,000 | Proteintech | 51153-1-AP |
| Phospho-JNK (Tyr185) | WB, 1:1,000 | Proteintech | 80024-1-RR |
| c-Jun | WB, 1:1,000 | Proteintech | 24909-1-AP |
| Phospho-c-Jun | WB, 1:1,000 | Proteintech | 80086-1-RR |
| Cyclin D1 | WB, 1:5,000 | Proteintech | 60186-1-Ig |
| CDK6 | WB, 1:1,000 | Proteintech | 14052-1-AP |
| K63-linkage-specific polyubiquitin | WB, 1:1,000 | Abcam | ab179434 |
| K48-linkage-specific polyubiquitin | WB, 1:1,000 | Abcam | ab140601 |
| Flag/DYKDDDDK tag | WB, 1:1,000; IP, 2 μg/IP | Proteintech | 66008-4-Ig |
| HA tag | WB, 1:1,000 | Proteintech | 51064-2-AP |
| Tubulin | WB, 1:10,000 | Proteintech | 66031-1-Ig |
| β-actin | WB, 1:10,000 | Proteintech | 66009-1-Ig |
| Vinculin | WB, 1:10,000 | Proteintech | 26520-1-AP |
| HRP-conjugated anti-mouse IgG | WB, 1:5,000 | Proteintech | SA00001-1 |
| HRP-conjugated anti-rabbit IgG | WB, 1:5,000 | Proteintech | SA00001-2 |

**Part B.** Key reagents

| **Reagent** | **Supplier** | **Catalogue No.** |
| --- | --- | --- |
| RIPA lysis buffer | Beyotime | P0013B |
| Protease and phosphatase inhibitor | Beyotime | P1045 |
| BCA Protein Assay Kit | Beyotime | P0012 |
| ECL reagent | Biosharp | BL523A |
| RNA-Quick Purification Kit | Yishan | ES-RN001 |
| Fast All-in-One RT Kit | Yishan | ES-RT001 |
| Super SYBR Green qPCR Master Mix | Yishan | ES-QP002 |
| CCK-8 | Beyotime | C003 |
| Classic Magnetic Protein A/G IP/Co-IP Kit | Yamei | YJ201 |
| Cycloheximide | MedChemExpress | HY-12320 |
| MG132 | MedChemExpress | HY-13259 |
| Lipofectamine 3000 | Thermo Fisher Scientific | L3000075 |
| DMSO | TargetMol | T0341 |
| SP600125 | TargetMol | T3109 |
