# Supplementary material for: UBE2S promotes gastric cancer progression through RACGAP1 stabilization associated with K292-dependent K63-linked ubiquitination
Source: Cancer Biol Ther. 2026 Oct 8;27(1):2737476. doi: 10.1080/15384047.2026.2737476 (PMC13652055; doi:10.1080/15384047.2026.2737476)
Supplement: Supplementary file 3 [file KCBT_A_2737476_SM7794.docx]

**Table S1. The sequences for primers in RT-qPCR**

| Genes |  | **Sequences (5’-3’)** |
| --- | --- | --- |
| UBE2S | Forward | ACAAGGAGGTGACGACACTGA |
|  | Reverse | CCACGTTCGGGTGGAAGAT |
| RACGAP1 | Forward | CTTTCCTTGCCTCTGGAGTATT |
|  | Reverse | ATCTGGTGTCTGTGGTGTTG |
| GAPDH | Forward | CCTGCACCACCAACTGCTTA |
|  | Reverse | TGAGTCCTTCCACGATACCA |
